# Supplementary material for: Characterization and Genomic Analysis of Fererhizobium litorale gen. nov., sp. nov., Isolated from the Sandy Sediments of the Sea of Japan Seashore
Source: Microorganisms. 2023 Sep 25;11(10):2385. doi: 10.3390/microorganisms11102385 (PMC10609224; doi:10.3390/microorganisms11102385)
Supplement: Supplementary file 1 [file microorganisms-11-02385-s001.zip › Supplementary Figures and Tables.pdf]

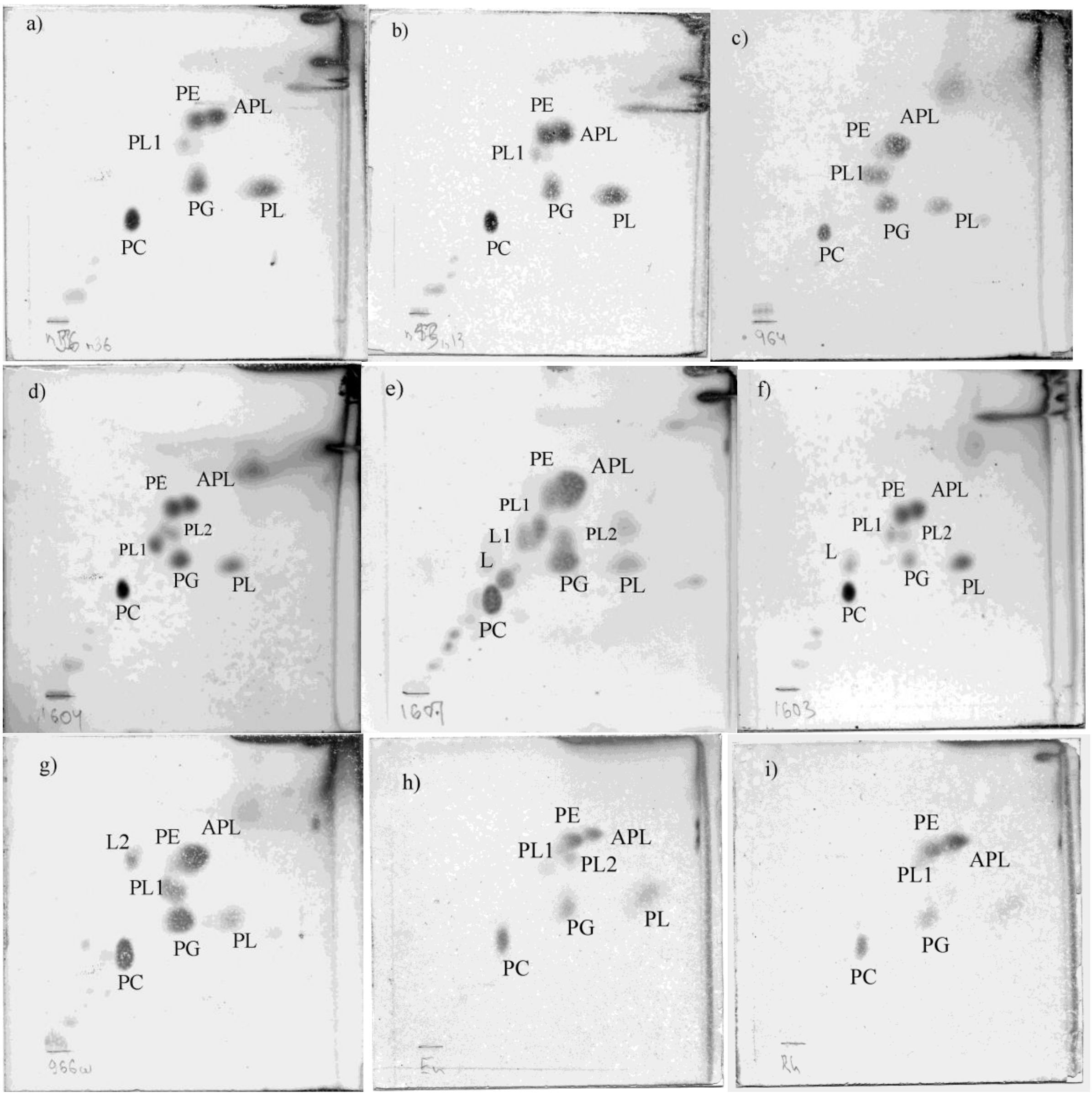

Figure S1. Two-dimensional thin-layer chromatograms of polar lipids of strains: (a) KMM 9576^{T}; (b) KMM 9553; (c) *Rhizobium leguminosarum* NBRC 14778^{T}; (d) *Agrobacterium radiobacter* NBRC 13532^{T}; (e) *Rhizobium rhizogenes* NBRC 13257^{T}; (f) *Sinorhizobium garamanticum* LMG 24692^{T}; (g) *Mesorhizobium mediterraneum* JCM 21565^{T}; (h) *Ensifer adhaerens* NBRC 100388^{T}; (i) *Pararhizobium giardinii* NBRC 107135^{T}. Abbreviations: PC, phosphatidylcholine, PE, phosphatidylethanoamine; PG, phosphatidylglycerol; APL, an unknown aminophospholipid; PL, PL1, PL2, unknown phospholipids; L, L1, L2, unknown lipids.

Table S1. Average nucleotide identity (ANI), average amino acid identity (AAI), and digital DNA-DNA hybridization (dDDH) values for studied strains KMM 9576^T and KMM 9553 and selected taxa from the family *Rhizobiaceae.*

**Genome 1**	**Genome 2**	**ANI, %**	**AAI, %**	**dDDH, %**
KMM 9576^T	KMM 9553	99.95	99.69	99.6
KMM 9576^T/ KMM 9553	*Rhizobium leguminosarum* USDA 2370^T	79.23/ 79.18	69.56/ 69.38	20.7/ 20.7
KMM 9576^T/ KMM 9553	*Neorhizobium galegae* HAMBI 540^T	78.85/ 78.81	67.78/ 67.72	20.8/ 20.8
KMM 9576^T/ KMM 9553	*Endobacterium cereale* RZME27^T	78.35/ 78.11	66.79/ 66.66	20.1/ 20.1
KMM 9576^T/ KMM 9553	*Pseudorhizobium pelagicum* R1-200B4^T	79.06/ 79.02	68.97/ 68.74	20.3/ 20.3
KMM 9576^T/ KMM 9553	*Agrobacterium radiobacter* CCUG 3354	78.59/ 78.60	68.07/ 67.87	20.4/ 20.4
KMM 9576^T/ KMM 9553	*Peteryoungia ipomoeae* shin9-1^T	79.14/ 79.10	68.23/ 68.06	20.3/ 20.4
KMM 9576^T/ KMM 9553	*Ciceribacter lividus* DSM 25528^T	79.26/ 79.23	69.84/ 69.66	20.4/ 20.4
KMM 9576^T/ KMM 9553	*Gellertiella hungarica* DSM 29853^T	79.43/ 79.32	67.10/ 66.79	20.4/ 20.4
KMM 9576^T/ KMM 9553	*Ensifer adhaerens* Casida A^T	79.87/ 79.90	69.18/ 69.06	21.6/ 21.6
KMM 9576^T/ KMM 9553	*Sinorhizobium fredii* USDA 205^T	79.59/ 79.49	68.75/ 68.60	21.0/ 21.0
KMM 9576^T/ KMM 9553	*Pararhizobium giardinii* H152^T	79.36/ 79.28	69.10/ 69.09	20.8/ 20.8
KMM 9576^T/ KMM 9553	*Shinella granuli* DSM 18401^T	79.66/ 79.70	69.09/ 68.90	21.1/ 21.1

Table S3. Differential physiological characteristics of strains KMM 9576^T and KMM 9553 and the most closely related bacteria. Strains: **1**, KMM 9576^T; **2**, KMM 9553; **3**, *Rhizobium leguminosarum* NBRC 14778^T; **4**, *Ensifer adhaerens* NBRC 100388^T; **5**, *Sinorhizobium garamanticum* LMG 24692^T; **6**, *Pararhizobium giardinii* NBRC 107135^T (data were obtained from the present study). All strains were positive for utilization of esculin ferric citrate and negative for utilization of glycerol, erythritol, dulcitol, inositol, D-mannitol, methyl-αD-mannopyranoside, methyl-αD-glucopyranoside, N-acetylglucosamine, amygdalin, salicin, D-lactose, inulin, amidon, glycogen, xylitol, D-turanose D-lyxose, D-tagatose, potassium gluconate, potassium 2-ketogluconate, potassium 5-ketogluconate.

**Utilization of:**	**1**	**2**	**3**	**4**	**5**	**6**
D-arabinose	(+)	+	-	+	+	-
L-arabinose	-	+	-	+	+	-
D-ribose	(+)	+	-	-	-	-
D-xylose	-	(+)	-	-	-	-
L-xylose	(+)	-	+	-	-	-
D-adonitol	-	-	-	+	+	-
Methyl-βD-xylopyranoside	-	-	-	+	-	-
D-galactose	-	-	-	+	+	-
D-glucose	-	-	-	+	+	-
D-fructose	-	-	-	+	+	-
D-mannose	-	-	-	+	+	-
L-sorbose	-	-	-	(+)	-	-
L-rhamnose	(+)	+	(+)	+	+	-
D-sorbitol	-	+	-	-	-	-
Arbutin	-	-	-	(+)	+	-
D-cellobiose	-	(+)	-	-	+	-
D-maltose	-	(+)	-	-	+	-
D-melibiose	-	(+)	-	+	+	-
D-saccharose	-	(+)	-	+	+	-
D-tregalose	-	(+)	-	-	-	-
D-melezitose	-	-	-	+	+	-
D-raffinose	-	-	-	(+)	+	-
Gentiobiose	-	(+)	-	+	-	-

D-fucose	+	+	+	+	-	-
L-fucose	+	-	-	+	-	-
D-arabitol	-	(+)	-	+	-	-
L-arabitol	-	-	-	+	-	-
